# Changes in Bacterial Communities and Metabolites Reveal the Effects of Starter Feeding on Growth Performance and Gut Development in Yak Calves

**DOI:** 10.3390/ani16132043

**Published:** 2026-07-02

**Authors:** Xinya Bie, Jinquan Yuan, Wenjie Guo, Yanan Zhou, Shujie Liu, Zhian Zhang, Xun Wang, Lu Sun, Jiaying Lv, Zhanhong Cui

**Affiliations:** 1 Qinghai Academy of Animal Husbandry and Veterinary Sciences, Qinghai University, Xining 810016, China; biexinya0304@126.com (X.B.); yjq13147637931@163.com (J.Y.); nyz0801@126.com (W.G.); yanan_zhou0312@163.com (Y.Z.);; 2 Key Laboratory of Plateau Grazing Animal Nutrition and Feed Science of Qinghai Province, Xining 810016, China; 3 Yak Engineering Technology Research Center of Qinghai Province, Xining 810016, China; 4 Ministry of Agriculture and Rural Affairs Key Laboratory of Animal Nutrition and Forage-Feed of Grazing Yak and Tibetan Sheep in Qinghai-Tibetan Plateau, Xining 810016, China

**Keywords:** starter feeding, yak calves, growth performance, gut development, bacterial community, metabolomics

## Abstract

This study investigated the effects of starter feed supplementation on growth performance, immune-related parameters, intestinal microbiota, and metabolite profiles in preweaning yak calves. Twenty healthy 30-day-old male yak calves with similar body weight were randomly assigned to either a control group (A), receiving milk replacer and alfalfa hay, or a starter-supplemented group (AS), receiving milk replacer, alfalfa hay, and a concentrate-based starter feed. Growth traits, nutrient digestibility, serum immune indices, intestinal microbial communities, and metabolomic characteristics were evaluated. Compared with the A group, calves in the AS group exhibited greater total dry matter intake, final body weight, heart girth, cannon circumference, and apparent digestibility of calcium and phosphorus (*p* < 0.05). Average daily gain tended to increase but did not differ significantly between treatments (*p* > 0.05). Starter supplementation also increased serum concentrations of IgA, IL-6, TNF-α, M-CSF, and IFN-γ (*p* < 0.05). In contrast, jejunal TNF-α concentration was lower in the AS group than in the A group (*p* < 0.05). Microbial analysis demonstrated that starter supplementation modified intestinal bacterial community composition. In the jejunum, the relative abundances of *Family_XIII_AD3011_group* and *Acetitomaculum* were increased, whereas *Bacteroidota* and *Bacteroides* were enriched in the colon. Untargeted metabolomic analysis further revealed distinct metabolic profiles between groups, with differential metabolites mainly associated with amino acid metabolism, vitamin metabolism, and energy-related pathways. Overall, starter feed supplementation was associated with changes in nutrient utilization, immune-related indicators, intestinal microbial composition, and metabolic characteristics. These findings suggest that starter supplementation may facilitate dietary adaptation and support gastrointestinal function in preweaning yak calves.

## Simple Summary

Nutritional management during early life is critical for the growth and development of yak calves on the Qinghai–Tibet Plateau. In this study, calves fed milk replacer and alfalfa hay were supplemented with a concentrate-based starter feed to evaluate its effects on growth performance and gastrointestinal function. Starter supplementation increased feed intake, body weight, and nutrient utilization and was associated with changes in gut microbial composition and immune-related indicators. These results suggest that the provision of starter feed during the preweaning period may be an effective feeding strategy for improving calf performance and supporting gastrointestinal health.

## 1. Introduction

Yaks (*Bos grunniens*) are the predominant livestock species on the Qinghai–Tibet Plateau and provide essential ecological, economic, and social benefits for local pastoral communities [1,2]. The preweaning period is a critical stage in calf development because nutritional status during early life can influence subsequent growth performance, health status, and productive potential. However, yak calves are frequently exposed to nutritional challenges under plateau production conditions. Seasonal forage shortages, declining forage quality during the cold season, and reduced milk production of dams during late lactation often limit nutrient intake and compromise calf growth [3]. Insufficient nutrient supply may also impair immune function and increase the risk of disease and mortality, resulting in substantial economic losses for yak production systems [4]. Therefore, the development of effective nutritional strategies for preweaning calves is important for improving production efficiency and promoting sustainable development of the yak industry. Nutritional interventions during early life have attracted considerable attention in ruminant production. Previous studies have demonstrated that milk replacer feeding and supplementation with forage or concentrate feeds can improve feed intake, growth performance, and rumen development in young yak calves [5,6,7]. Starter feeds are typically formulated from highly digestible concentrate ingredients and are widely used to facilitate the transition from a milk-based diet to solid feed. In young ruminants, starter supplementation has been associated with improvements in feed intake, rumen fermentation, microbial colonization, and epithelial development [8]. Nevertheless, information regarding its effects on the intestinal environment of yak calves remains limited. The gastrointestinal tract serves not only as the primary site of nutrient digestion and absorption but also as an important interface between the host and its resident microbiota. Establishment of the intestinal microbial community during early life plays a key role in nutrient metabolism, immune regulation, and gastrointestinal function [9]. Through fermentation of dietary substrates, intestinal microorganisms generate metabolites such as short-chain fatty acids that contribute to host energy metabolism and physiological regulation [10,11]. In addition, interactions between gut microbiota and the intestinal immune system are essential for the maintenance of mucosal homeostasis and immune competence [12]. Alterations in microbial composition may therefore influence both metabolic activity and immune responses within the gastrointestinal tract. Although the effects of starter supplementation on growth performance and rumen development have been extensively investigated, its influence on intestinal microbial ecology, metabolic characteristics, and immune-related responses in yak calves remains poorly understood. Therefore, the present study combined 16S rRNA gene sequencing with untargeted metabolomics to evaluate the effects of starter feed supplementation on intestinal microbiota composition, metabolite profiles, immune-related parameters, and gastrointestinal function in preweaning yak calves. We hypothesized that starter supplementation would modify intestinal microbial and metabolic characteristics and thereby contribute to gastrointestinal adaptation during early life. The findings provide further insight into the microbiota–metabolite interactions associated with starter feeding under plateau production conditions.

## 2. Materials and Methods

### 2.1. Experimental Animals and Group Design

Twenty clinically healthy male yak calves (30 d of age; body weight 31.60 ± 1.12 kg) were selected from Datong Breeding Farm, Qinghai Province, China. All calves were transported to the Plateau Ecological Animal Husbandry Science and Technology Demonstration Park for the feeding experiment. During the first 30 d postpartum, calves received colostrum promptly and were subsequently managed under a system of natural suckling combined with grazing. Following a 30 d adaptation period, animals were randomly allocated into two treatment groups (n = 10 per group): (1) control group (A), receiving milk replacer and alfalfa hay and (2) starter-fed group (AS), receiving milk replacer, alfalfa hay, and starter concentrate. The experiment consisted of a 30 d adaptation phase and a 100 d formal feeding trial (15 August to 22 November 2020). At the end of the trial, five calves per group were randomly selected for sample collection, including blood and intestinal tissues for subsequent analyses of morphology, digestive enzyme activity, immune parameters, microbial community profiling, and metabolomic characterization.

### 2.2. Experimental Site and Ethical Approval

The experiment was conducted at the Plateau Ecological Animal Husbandry Science and Technology Demonstration Park, Haibei Tibetan Autonomous Prefecture, Qinghai Province, China (100°96′ E, 36°92′ N; altitude 3010 m; mean annual temperature 1.5 °C). All procedures involving animals were approved by the Animal Ethics Committee of the Qinghai Academy of Animal Husbandry and Veterinary Sciences (Approval No. 2025-QHMKY-022) and complied with national guidelines for experimental animal welfare. Sample analyses were performed at the Key Laboratory of Nutrition and Feed Science for Plateau Grazing Animals, Qinghai University, and the Qinghai Academy of Animal Husbandry and Veterinary Sciences.

### 2.3. Experimental Diets and Feeding Management

All calves (*n* = 20) were used for measurements of feed intake, growth performance, body size indices, and nutrient digestibility. At the end of the 100 d trial, five calves per group were selected for blood sampling and slaughter to obtain intestinal tissues for subsequent analyses. Calves in group A were fed milk replacer and alfalfa hay, whereas calves in group AS received milk replacer, alfalfa hay, and starter concentrate. Milk replacer was provided at equal dry matter intake across treatments. Solid feed provision (alfalfa hay in group A and alfalfa hay plus starter in group AS) was designed to maintain comparable total solid feed dry matter intake between groups. Calves were housed individually and fed twice daily at 08:00 and 16:00 h. Milk replacer was reconstituted with warm water (42 °C) at a 1:5 (*w*/*v*) ratio and administered via bottle feeding. Fresh water was freely available throughout the experimental period. From days 1 to 50, alfalfa hay and starter feed in group AS were offered at a dry matter ratio of 2:1, which was adjusted to 1:1 during days 51 to 100 in accordance with growth stage and feed intake patterns. Daily feed offered and refusals were recorded to calculate dry matter intake. A digestibility trial was conducted during the final phase of the experiment using the total fecal collection method. Feces were collected for five consecutive days at 08:00 h, immediately weighed, and acidified to minimize nitrogen loss. Composite fecal samples were prepared for each animal based on daily output and used for subsequent nutrient analyses. Body weight and structural body measurements were recorded at the end of the experiment. The chemical composition of milk replacer, alfalfa hay, and starter feed is presented in Table 1 and Table 2. The starter concentrate was formulated primarily from corn (46.5%), soybean meal (27.0%), wheat bran (9.5%), extruded soybean meal (8.0%), and dried whey (5.0%) on a dry matter basis. Minor ingredients included limestone, dicalcium phosphate, sodium chloride, fat powder, and a vitamin–mineral premix. The starter feed contained 24.1% crude protein, 3.74% ether extract, 10.9% neutral detergent fiber (NDF), 4.1% acid detergent fiber (ADF), 0.80% calcium, and 0.45% phosphorus. The fiber fraction was primarily derived from wheat bran and soybean-based components. Feed intake is shown in Table 3.

### 2.4. Sample Collection

After completion of the 100-day feeding trial, five clinically healthy calves were randomly selected from each treatment group for sample collection. Prior to the morning feeding, jugular blood samples were obtained and body weight and morphometric measurements were recorded. Calves were anesthetized by intravenous injection of 10% chloral hydrate solution (100 mg/kg body weight; Sigma-Aldrich, St. Louis, MO, USA) prior to exsanguination and tissue collection. The gastrointestinal tract was removed, and the jejunum and colon were separated. Digesta samples were aseptically collected from the anterior portions of the jejunum and colon, immediately frozen in liquid nitrogen, and stored at −80 °C for subsequent analyses of digestive enzyme activity, microbial community composition, and metabolomic profiles. Following removal of intestinal contents, the jejunum and colon were weighed. Tissue samples from the anterior jejunum and colon were fixed in 4% paraformaldehyde for histomorphological evaluation. Intestinal mucosal samples were collected by gentle scraping with sterile glass slides, snap-frozen in liquid nitrogen, and stored at −80 °C for subsequent determination of immune-related parameters.

### 2.5. Measurement Indexes and Methods

#### 2.5.1. Measurement of Plasma Biochemical and Immune Indexes

Plasma biochemical parameters, including total protein (TP), albumin (ALB), alanine aminotransferase (ALT), aspartate aminotransferase (AST), globulin (GLO), glucose (GLU), total cholesterol (CHOL), blood urea nitrogen (BUN), creatinine (CRE), triglycerides (TG), lactate dehydrogenase (LDH), alkaline phosphatase (ALP), calcium (Ca), and phosphorus (P), were determined using an automated biochemical analyzer (Cobas 601, Roche Diagnostics, Mannheim, Germany). Plasma concentrations of interleukin-2 (IL-2), IL-4, IL-6, IL-10, immunoglobulin A (IgA), immunoglobulin G (IgG), immunoglobulin M (IgM), tumor necrosis factor-α (TNF-α), macrophage colony-stimulating factor (M-CSF), interferon-α (IFN-α), IFN-β, and IFN-γ were quantified using commercial bovine-specific ELISA kits (Jiangsu Enzyme Biotechnology Co., Ltd., Yancheng, China) according to the manufacturer’s instructions. Absorbance was measured at 450 nm using a microplate reader (Synergy 2, BioTek Instruments, Winooski, VT, USA), and concentrations were calculated from standard curves generated for each analyte. Given the close phylogenetic relationship between yak (*Bos grunniens*) and cattle (*Bos taurus*), bovine-specific ELISA kits have been extensively used in previous studies involving yak biological samples. The intra-assay and inter-assay coefficients of variation provided by the manufacturer were <10% and <15%, respectively. Assay sensitivities complied with the manufacturer’s specifications for all analytes.

#### 2.5.2. Determination of Gut Morphology, Digestive Enzyme Activity and Immune Factor Content

Jejunal and colonic tissue samples were fixed in 4% paraformaldehyde, dehydrated through a graded ethanol series, cleared in xylene, embedded in paraffin, and sectioned at a thickness of 5 μm using a rotary microtome (RM2235, Leica Microsystems, Wetzlar, Germany). Sections were stained with hematoxylin and eosin (H&E) for histomorphological evaluation. For each sample, three non-consecutive longitudinal sections were prepared. Sections were examined under a light microscope (Olympus TH4-200, Tokyo, Japan), and five randomly selected microscopic fields were photographed from each section. Morphological measurements were performed using cellSens Entry software (version 1.18, Olympus, Tokyo, Japan). For each image, 3–5 measurements of the corresponding morphological parameters were obtained, and the average value was used for statistical analysis. Villus height (VH), crypt depth (CD), and the villus height-to-crypt depth ratio (VH/CD) were measured in the jejunum, whereas mucosal thickness and crypt depth were measured in the colon. The activities of trypsin, chymotrypsin, α-amylase, and lipase in the jejunum, as well as α-amylase, lipase, and cellulase in the colon, were determined using commercial ELISA kits (Jiangsu Enzyme Biotechnology Co., Ltd., Yancheng, China). Concentrations of secretory immunoglobulin A (sIgA), IL-2, IL-6, IL-10, TNF-α, and IFN-γ in jejunal and colonic mucosal samples were also quantified using ELISA according to the manufacturer’s instructions. Prior to analysis, intestinal contents and mucosal samples were processed as tissue homogenates. Briefly, intestinal contents were diluted with sterile physiological saline (0.9% NaCl) at a ratio of 1:9 (*w*/*v*), homogenized thoroughly, and centrifuged at 2500 rpm for 20 min at 4 °C. The resulting supernatants were collected for subsequent analyses. Intestinal mucosal samples were ground in liquid nitrogen and homogenized in physiological saline using the same dilution ratio and centrifugation procedure. The supernatants were then used for enzyme activity and immune factor determination. According to the manufacturer, the intra-assay and inter-assay coefficients of variation were <10% and <15%, respectively. Assay sensitivities were <1.0 U/L for trypsin, <1.0 IU/L for chymotrypsin, α-amylase, lipase, and cellulase, and <0.1 μg/mL for sIgA.

#### 2.5.3. Microbial DNA Extraction, 16S rRNA Gene Amplification and Bioinformatics Analysis

Total genomic DNA was extracted from jejunal and colonic digesta samples using the CTAB/SDS method. DNA concentration and purity were assessed by agarose gel electrophoresis (1%) and spectrophotometric analysis. DNA samples were diluted to 1 ng/μL with sterile water prior to PCR amplification. The V3–V4 hypervariable region of the bacterial 16S rRNA gene was amplified using the universal primers 341F (5′-CCTAYGGGRBGCASCAG-3′) and 806R (5′-GGACTACHVGGGTWTCTAAT-3′). Each PCR reaction contained 15 μL Phusion^®^ High-Fidelity PCR Master Mix (New England Biolabs, Ipswich, MA, USA), 0.2 μM of each primer, and approximately 10 ng template DNA. Amplification was performed under the following conditions: initial denaturation at 98 °C for 1 min, followed by 30 cycles of 98 °C for 10 s, 50 °C for 30 s, and 72 °C for 30 s, with a final extension at 72 °C for 5 min. PCR products were mixed with loading buffer, separated by electrophoresis on 2% agarose gels, and purified using a QIAquick Gel Extraction Kit (Qiagen, Hilden, Germany). Sequencing libraries were prepared using the TruSeq^®^ DNA PCR-Free Sample Preparation Kit (Illumina, San Diego, CA, USA) following the manufacturer’s protocol, and index codes were added to individual samples. Library quality was assessed using a Qubit^®^ 2.0 Fluorometer (Thermo Fisher Scientific, Waltham, MA, USA) and an Agilent 2100 Bioanalyzer (Agilent Technologies, Santa Clara, CA, USA). Paired-end sequencing (2 × 250 bp) was subsequently performed on an Illumina NovaSeq platform. Raw paired-end reads were assigned to samples according to unique barcode sequences and merged using FLASH software (v1.2.7) [13]. Quality filtering was conducted using QIIME (v1.9.1) to obtain high-quality clean reads [14,15]. Chimeric sequences were identified by comparison with the reference database and removed to generate effective tags [16]. Sequences were clustered into operational taxonomic units (OTUs) at 97% sequence similarity using UPARSE (v7.0.1001), and representative sequences were selected for downstream analyses [17]. Taxonomic classification was performed using the Mothur algorithm against the SILVA 132 SSU rRNA database [18,19]. Community composition at the phylum and genus levels was determined based on taxonomic annotations. For diversity analyses, sequence data were rarefied to the same sequencing depth across all samples. Alpha-diversity indices, including Good’s coverage, Chao1, ACE, Shannon, and Simpson indices, were calculated, and beta-diversity analyses were performed based on Bray–Curtis distance matrices. For comparisons of bacterial taxa between treatment groups, relative abundance data at the phylum and genus levels were analyzed using Student’s *t*-test. To reduce the risk of false-positive results arising from multiple comparisons, *p*-values were adjusted using the Benjamini–Hochberg false discovery rate (FDR) procedure. Differences were considered statistically significant at FDR-adjusted *p* < 0.05.

#### 2.5.4. Metabolomics Analysis

Jejunal and colonic samples were processed for untargeted metabolomic profiling. Briefly, 100 μL of each sample was mixed with pre-chilled 80% methanol containing 0.1% formic acid and vortexed thoroughly. After incubation on ice for 5 min, samples were centrifuged at 15,000× *g* for 20 min at 4 °C. A portion of the resulting supernatant was diluted with LC–MS-grade water to obtain a final methanol concentration of 53%. The diluted extracts were transferred to fresh microcentrifuge tubes and centrifuged again at 15,000× *g* for 20 min at 4 °C. The final supernatants were subjected to UHPLC–MS/MS analysis. Metabolomic analyses were performed by Novogene Co., Ltd. (Beijing, China) using a Vanquish UHPLC system coupled to an Orbitrap Q Exactive™ HF-X mass spectrometer (Thermo Fisher Scientific, Waltham, MA, USA). Samples were separated on a Hypersil Gold column (100 mm × 2.1 mm, 1.9 μm particle size) using a 17 min linear gradient at a flow rate of 0.2 mL/min. For positive ion mode, mobile phase A consisted of water containing 0.1% formic acid and mobile phase B consisted of methanol. For negative ion mode, mobile phase A consisted of 5 mM ammonium acetate (pH 9.0), whereas methanol served as mobile phase B. The solvent gradient was programmed as follows: 2% B for 1.5 min, 2–100% B from 1.5 to 12.0 min, 100% B until 14.0 min, 100–2% B from 14.0 to 14.1 min, followed by equilibration at 2% B until 17.0 min. The mass spectrometer was operated in both positive and negative electrospray ionization modes with a spray voltage of 3.2 kV, capillary temperature of 320 °C, sheath gas flow rate of 40 arb, and auxiliary gas flow rate of 10 arb. Raw LC–MS/MS data were processed using Compound Discoverer 3.1 (Thermo Fisher Scientific) for peak alignment, peak detection, and metabolite quantification. Parameters included a retention time tolerance of 0.2 min, mass tolerance of 5 ppm, signal intensity tolerance of 30%, signal-to-noise ratio of 3, and minimum intensity threshold settings according to the software recommendations. Peak intensities were normalized to the total spectral intensity. Metabolite identification was performed by matching molecular ions, adduct ions, and fragment ions against the mzCloud, mzVault, and MassList databases. Quality control (QC) samples were generated by pooling equal aliquots of all biological samples and were analyzed throughout the analytical sequence to evaluate instrument stability and reproducibility. Pearson correlation coefficients among QC samples were calculated, and high correlations (R^2^ values approaching 1.0) indicated excellent analytical stability and data reliability. Metabolites were further annotated using the Kyoto Encyclopedia of Genes and Genomes (KEGG), Human Metabolome Database (HMDB), and LIPID MAPS databases. Differential metabolites between groups were identified based on variable importance in projection (VIP) values derived from partial least squares discriminant analysis (PLS-DA), fold change (FC), and univariate statistical testing. Metabolites with VIP > 1.0, *p* < 0.05, and FC ≥ 1.5 or FC ≤ 0.667 were considered differentially abundant. To assess model robustness and avoid overfitting, permutation testing was performed for PLS-DA model validation.

### 2.6. Statistical Analysis

Statistical analyses were performed using SPSS 26.0 (IBM Corp., Armonk, NY, USA), and figures were generated using GraphPad Prism 9 (GraphPad Software, San Diego, CA, USA). Prior to analysis, all data were tested for normality using the Shapiro–Wilk test and for homogeneity of variance using Levene’s test. Data meeting these assumptions were analyzed using Student’s *t*-test. Results are presented as mean ± SEM, and statistical significance was declared at *p* < 0.05. For microbiome analyses, alpha-diversity indices, including Good’s coverage, ACE, Chao1, Shannon, and Simpson indices, were calculated using QIIME (v1.9.1). Differences in OTU numbers, alpha-diversity indices, and bacterial relative abundances at the phylum and genus levels were evaluated using Student’s *t*-test. To control for multiple comparisons, *p*-values were adjusted using the Benjamini–Hochberg false discovery rate (FDR) procedure, and FDR-adjusted *p* < 0.05 was considered statistically significant. Beta-diversity was assessed using Bray–Curtis distance matrices, and differences in community structure between groups were evaluated using analysis of similarities (ANOSIM) implemented in the vegan package of R software (version 4.3.2). For metabolomic analyses, partial least squares discriminant analysis (PLS-DA) was performed using MetaX software (version 2.6.0) to calculate variable importance in projection (VIP) scores. Differential metabolites were identified based on the criteria of VIP > 1.0, *p* < 0.05 (Student’s *t*-test), and fold change (FC) ≥ 1.5 or ≤0.667. False discovery rate correction was not applied to the metabolomic dataset because stringent correction substantially reduced the number of detectable differential metabolites. Therefore, the metabolomic results should be interpreted with caution and regarded as exploratory. Functional annotation and pathway enrichment analyses of differential metabolites were conducted using the Kyoto Encyclopedia of Genes and Genomes (KEGG) database, and pathways with *p* < 0.05 were considered significantly enriched. To evaluate model robustness and avoid overfitting, PLS-DA models were validated using permutation testing. Negative Q^2^ intercept values obtained from permutation analyses indicated acceptable predictive performance and confirmed that the models were not overfitted.

## 3. Results

### 3.1. Characterization of Growth Performance of Yak Calves

**Figure 1:**
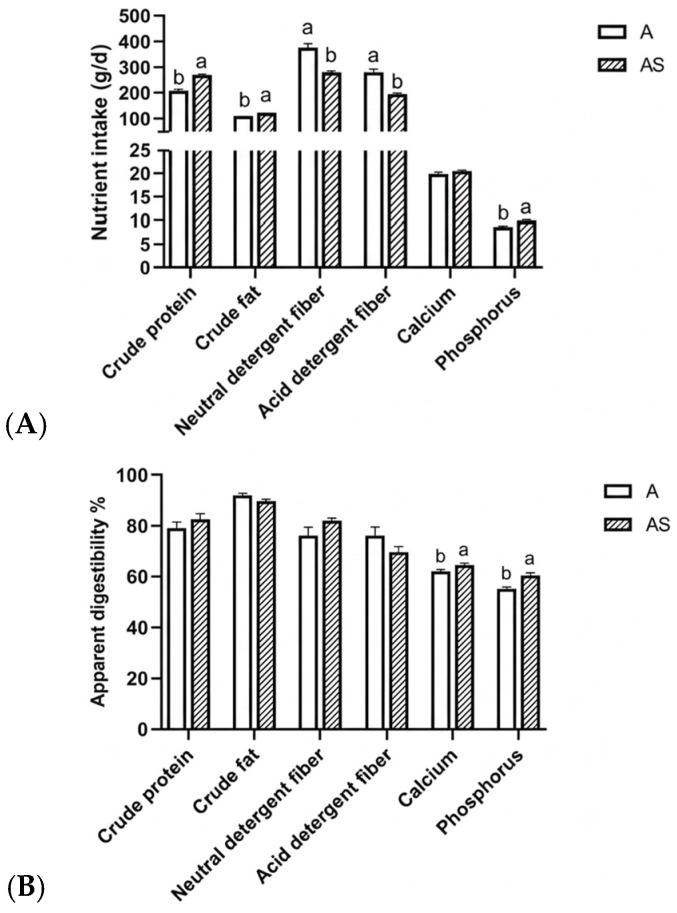
Effects of starter supplementation on nutrient intake and nutrient digestibility in yak calves. (**A**) Daily nutrient intake (**B**) Apparent nutrient digestibility. A, alfalfa hay group; AS, alfalfa hay plus starter feed group. The data shown as mean ± SEM were analyzed using *t*-test. Lowercase letters a and b are labeled to indicate a significant difference between the two groups (*p* < 0.05). No labeling indicates no significant difference between the two groups (*p* > 0.05). Note: Estimated daily metabolizable energy (ME) intake was 16.25 MJ/d in the A group and 18.29 MJ/d in the AS group, These values represent estimated rather than directly measured ME intake.

Compared with calves fed milk replacer and alfalfa hay alone, starter-supplemented calves showed higher final body weight, heart girth, and cannon bone circumference, with the increase in heart girth being highly significant (*p* < 0.001; Table 4). In contrast, no treatment effects were detected for average daily gain, feed conversion ratio, body height, or body length (*p* > 0.05). Dietary supplementation with starter feed markedly modified nutrient intake characteristics. Calves in the AS group consumed greater amounts of crude protein, ether extract, and phosphorus, whereas intake of neutral detergent fiber (NDF) and acid detergent fiber (ADF) were reduced relative to the control group (*p* < 0.05; Figure 1A). Moreover, apparent digestibility coefficients of calcium and phosphorus were significantly enhanced by starter supplementation (*p* < 0.05; Figure 1B). Evaluation of circulating biochemical and immune-related variables revealed that starter-fed calves exhibited elevated concentrations of IgA, IL-6, TNF-α, M-CSF, and IFN-γ compared with control calves (*p* < 0.05). Conversely, serum albumin levels were lower in the AS group than in the A group (*p* < 0.05; Table 5).

### 3.2. Characterization of Gut Development of Yak Calves

**Figure 2:**
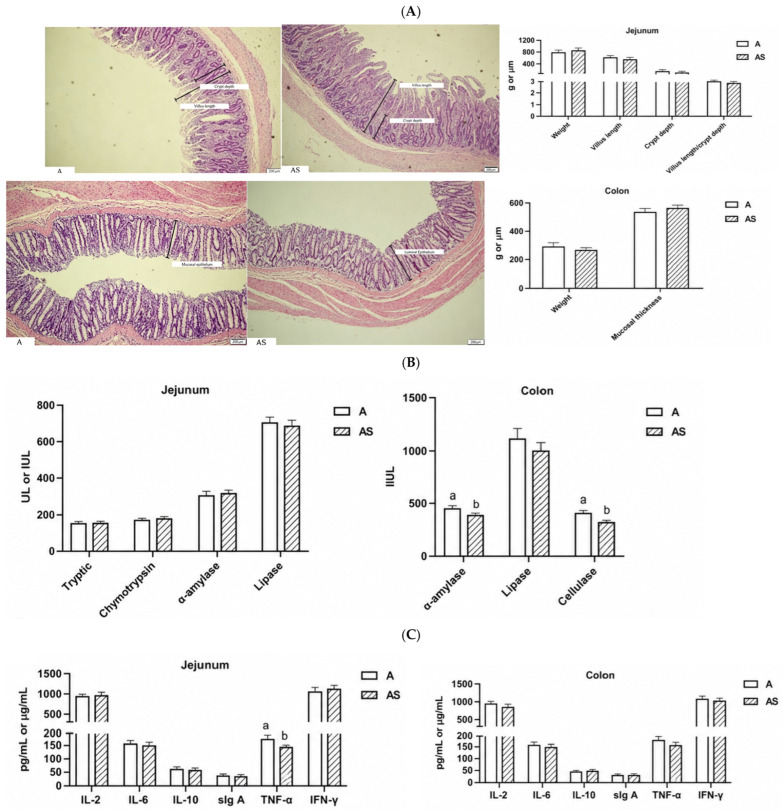
Effects of starter supplementation on intestinal development, digestive enzyme activities, and immune factors in yak calves. (**A**) Representative histological sections and morphometric measurements of the jejunum and colon (H&E staining; scale bar = 100 μm). (**B**) Digestive enzyme activities in the jejunum and colon. (**C**) Immune factor concentrations in the jejunum and colon. A, alfalfa hay group; AS, alfalfa hay plus starter feed group. IL-2, interleukin-2; IL-6, interleukin-6; IL-10, interleukin-10; SIgA, secretory immunoglobulin A; TNF-α, tumor necrosis factor-α; IFN-γ, interferon-γ. Values are presented as mean ± SEM. Different lowercase letters (a, b) above bars indicate significant differences between groups (*p* < 0.05).

No significant differences were detected between treatments in jejunal villus height, crypt depth, villus height-to-crypt depth ratio, or colonic mucosal thickness (*p* > 0.05; Figure 2A), indicating that starter supplementation had limited effects on intestinal morphological development during the experimental period. With respect to digestive enzyme activities, calves in the AS group exhibited significantly lower colonic α-amylase and cellulase activities than those in the A group (*p* < 0.05; Figure 2B). No significant treatment effects were observed for the remaining digestive enzymes (*p* > 0.05). Analysis of intestinal mucosal immune-related factors revealed that jejunal TNF-α concentration was significantly reduced in starter-supplemented calves compared with control calves (*p* < 0.05; Figure 2C), whereas no significant differences were detected for the other measured immune indicators (*p* > 0.05).

### 3.3. Diversity and Composition of Gut Bacterial Community

**Figure 3:**
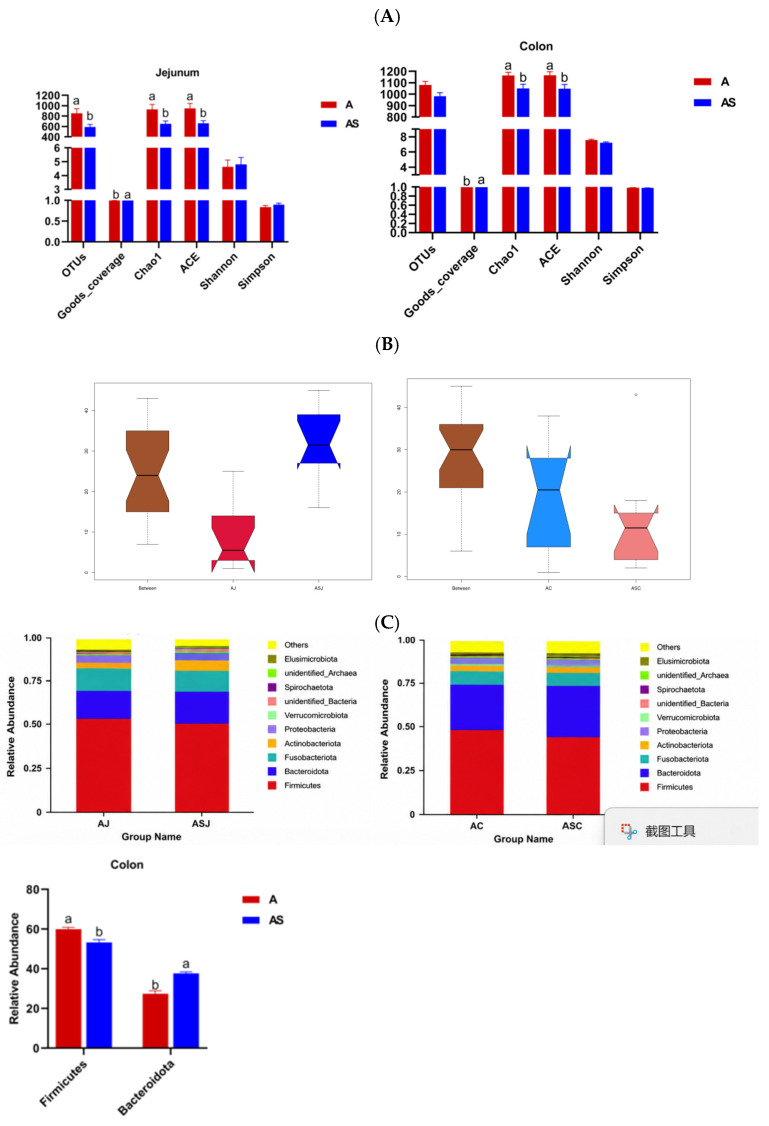
Effects of starter feed supplementation on intestinal bacterial community composition in yak calves. (**A**) OTU numbers and alpha-diversity indices. (**B**) ANOSIM analysis based on Bray–Curtis distance matrices. (**C**) Relative abundance of bacterial phyla. The Chinese text in (**C**) means screenshot tool. AJ, jejunum of calves in the A group; ASJ, jejunum of calves in the AS group; AC, colon of calves in the A group; ASC, colon of calves in the AS group. These abbreviations are used consistently throughout the subsequent figures.

The bacterial communities of the jejunum and colon were characterized by 16S rRNA gene sequencing. Good’s coverage values approached 1.0 for all samples (Figure 3A), indicating sufficient sequencing depth and comprehensive coverage of the bacterial populations. Compared with the A group, calves in the AS group exhibited significantly lower OTU numbers in the jejunum and reduced Chao1 and ACE indices in both intestinal segments (*p* < 0.05; Figure 3A), suggesting a decrease in bacterial richness following starter supplementation. However, changes in richness should not be interpreted as inherently beneficial or detrimental. Beta-diversity analysis based on ANOSIM revealed a significant separation of jejunal microbial communities between treatment groups (*p* < 0.05; Figure 3B), indicating that starter supplementation altered the overall bacterial community structure in the jejunum. At the phylum level (Figure 3C), *Firmicutes* predominated in the jejunum and did not differ significantly between treatments (*p* > 0.05). In the colon, *Firmicutes* and *Bacteroidota* were the most abundant phyla. Relative to the A group, calves receiving starter supplementation exhibited a higher abundance of *Bacteroidota* and a lower abundance of *Firmicutes* (*p* < 0.05). At the genus level (Table 6), *Romboutsia* was the predominant genus in the jejunum. Starter supplementation significantly reduced the relative abundances of *Olsenella*, *Turicibacter*, *Bacteroides*, and *Colidextribacter*, while increasing the abundances of *Family_XIII_AD3011_group* and *Acetitomaculum* (*p* < 0.05). Although *Akkermansia* tended to decrease in the AS group, the difference did not reach statistical significance (*p* > 0.05). In the colon, UCG-005 was the dominant genus across both treatments. Compared with calves in the A group, calves in the AS group showed a greater relative abundance of *Bacteroides* and lower relative abundances of *Christensenellaceae_R-7_group*, *Acetitomaculum*, and *Oribacterium* (*p* < 0.05). No significant differences were detected for the other major genera (*p* > 0.05).

### 3.4. Differential Metabolites and Differential Metabolic Pathways of the Gut

**Figure 4:**
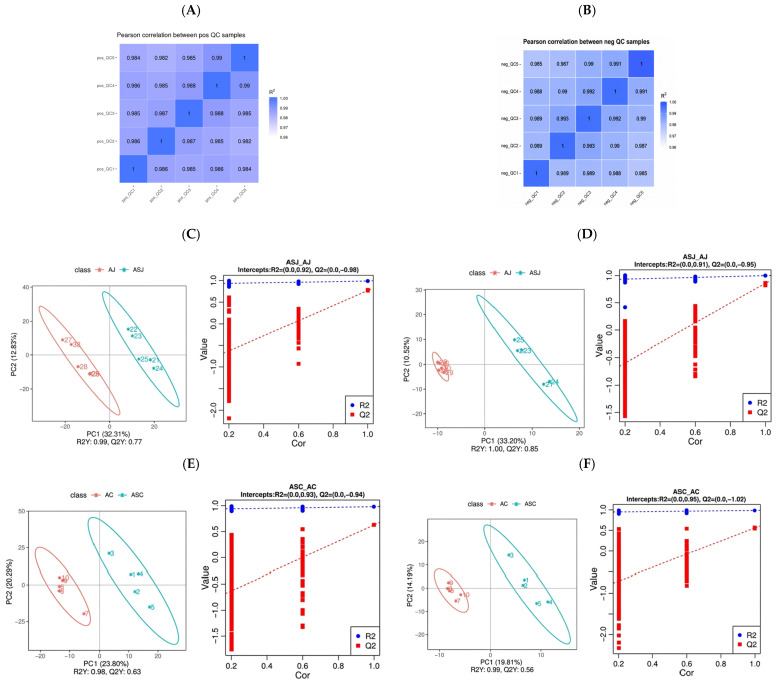
Quality control assessment and partial least squares discriminant analysis (PLS-DA) of intestinal metabolites in yak calves. (**A**) Pearson correlation coefficients among quality control (QC) samples in positive ion mode. (**B**) Pearson correlation coefficients among QC samples in negative ion mode. (**C**) PLS-DA score plot of jejunal metabolites in positive ion mode. (**D**) PLS-DA score plot of jejunal metabolites in negative ion mode. (**E**) PLS-DA score plot of colonic metabolites in positive ion mode. (**F**) PLS-DA score plot of colonic metabolites in negative ion mode. Note: In the permutation test plots, the blue and red dashed lines represent the regression lines of the permuted R^2^ and Q^2^ values, respectively.

**Figure 5:**
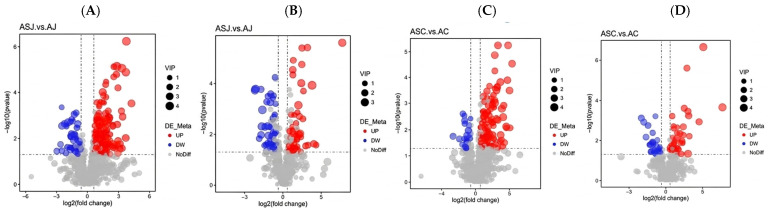
Volcano plots showing differential metabolites between the A and AS groups. (**A**) Jejunal metabolites detected in positive ion mode. (**B**) Jejunal metabolites detected in negative ion mode. (**C**) Colonic metabolites detected in positive ion mode. (**D**) Colonic metabolites detected in negative ion mode. Note: The vertical dashed lines indicate the thresholds of log2(fold change) = ±1, and the horizontal dashed line indicates the significance threshold of *P* = 0.05 [−log10(P) = 1.30].

**Figure 6:**
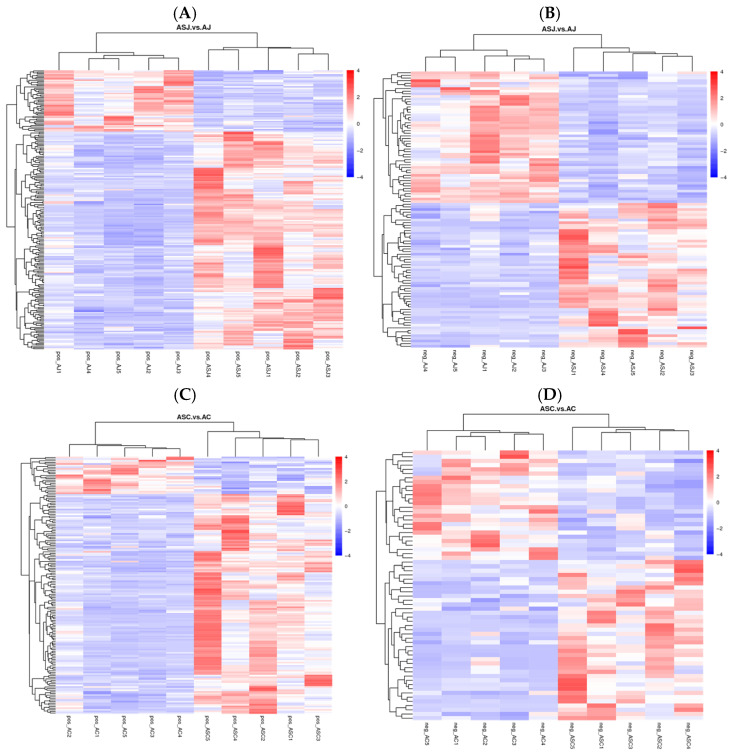
Hierarchical clustering analysis of differential metabolites in the jejunum and colon of yak calves. (**A**) Jejunal metabolites detected in positive ion mode. (**B**) Jejunal metabolites detected in negative ion mode. (**C**) Colonic metabolites detected in positive ion mode. (**D**) Colonic metabolites detected in negative ion mode. Samples and metabolites were clustered based on Euclidean distance, and metabolite abundance is represented by the color scale.

Quality control samples were incorporated throughout the analytical sequence to monitor instrument performance and ensure data reliability. Strong correlations among QC samples demonstrated satisfactory analytical consistency and reproducibility of the metabolomic platform (Figure 4A,B). Distinct metabolic patterns between treatment groups were further explored using PLS-DA. Cross-validation results indicated that the models possessed acceptable explanatory and predictive capacities (Figure 4C–F). Permutation tests confirmed model validity and suggested that overfitting was unlikely. Based on the predefined screening thresholds (VIP > 1.0, *p* < 0.05, and FC ≥ 1.5 or ≤0.667), numerous metabolites were identified as significantly altered by starter supplementation. Visualization using volcano plots revealed clear differences in metabolite abundance between groups (Figure 5). Hierarchical clustering further demonstrated consistent grouping of biological replicates and a clear separation of treatment-specific metabolic profiles (Figure 6). In the jejunum, 209 and 105 differential metabolites were detected under positive and negative ionization modes, respectively. Among these metabolites, the majority showed higher abundance in calves receiving starter supplementation. Similarly, 158 and 64 differential metabolites were identified in the colon under positive and negative ionization modes, respectively, with most metabolites exhibiting increased abundance in the AS group. KEGG enrichment analysis indicated that starter supplementation primarily influenced amino acid-related metabolic pathways in the jejunum, including aminoacyl-tRNA biosynthesis, phenylalanine, tyrosine and tryptophan biosynthesis, and alanine, aspartate and glutamate metabolism. In contrast, differential metabolites in the colon were mainly enriched in riboflavin metabolism, endocrine resistance, and lysine degradation pathways. Collectively, these findings suggest that starter supplementation was associated with substantial alterations in intestinal metabolic activity, particularly in pathways related to amino acid and vitamin metabolism (Table 7 and Table 8).

## 4. Discussion

### 4.1. Growth Performance and Serum Biochemical Parameters

In traditional grazing systems on the Qinghai–Tibet Plateau, yak calves rely primarily on maternal milk during early life. However, forage availability and nutrient quality decline substantially during the cold season, while milk production of yak dams decreases simultaneously, resulting in limited nutrient supply for calf growth and development. Under these conditions, milk replacer serves as an effective nutritional supplement by providing readily digestible protein, energy, and essential nutrients required for early development [20]. In addition to liquid feeding, early introduction of solid feed is considered essential for stimulating gastrointestinal development and facilitating the transition from a milk-based diet to solid feed consumption. Previous studies have shown that forage supplementation can increase dry matter intake and support growth in young ruminants [21,22]. Alfalfa hay, as a high-quality forage source, promotes rumen epithelial development and contributes to rumen health and microbial establishment [23]. Starter supplementation provides additional fermentable carbohydrates and protein, which may further enhance nutrient supply and support gastrointestinal functional development [24]. Moreover, the combined provision of forage and starter feed has been reported to improve nutrient utilization and facilitate adaptation to post-weaning feeding conditions [25,26]. In the present study, calves receiving starter supplementation exhibited greater total dry matter intake and crude protein intake than calves fed milk replacer and alfalfa hay alone. Correspondingly, final body weight, heart girth, and cannon bone circumference were increased in the AS group. However, average daily gain and feed conversion ratio did not differ significantly between treatments, suggesting that although starter supplementation improved nutrient intake and certain body development traits, its effects on growth efficiency during the experimental period were limited. Previous studies have demonstrated that increased dietary protein supply can enhance nitrogen retention and support tissue accretion in growing calves [27,28]. In the current study, starter supplementation also altered several circulating immune-related indicators. Nevertheless, these responses should be interpreted cautiously because cytokine concentrations may reflect developmental adaptation of the immune system, dietary transition, and microbial–host interactions rather than a direct enhancement of immune function. Therefore, the observed changes are more appropriately considered indicators of immune modulation during early-life nutritional adaptation. In addition, the higher intake of crude protein and dietary energy provided by the starter diet may have contributed to the observed differences in nutrient utilization and physiological development. Dietary nutrients not only support growth directly but also influence gastrointestinal fermentation characteristics and host–microbiota interactions, thereby affecting developmental processes in young ruminants [29].

### 4.2. Intestinal Digestive Enzyme Activity

No significant differences were observed in jejunal villus height, crypt depth, villus height-to-crypt depth ratio, or colonic mucosal thickness between treatments. These findings indicate that starter supplementation did not markedly affect intestinal morphological development during the experimental period. Therefore, the beneficial effects of starter feeding should not be attributed to changes in intestinal structure. Digestive enzyme activity is an important indicator of gastrointestinal functionality and nutrient utilization in young ruminants. Although the rumen is the primary site of microbial fermentation in mature ruminants, the small intestine remains an important site for nutrient digestion and absorption, particularly during the transition from a milk-based diet to solid feed in young calves. In addition, the large intestine serves as a secondary fermentation site where resident microorganisms contribute to nutrient metabolism and host health [30]. In the present study, starter supplementation did not significantly affect digestive enzyme activities in the jejunum. However, colonic α-amylase and cellulase activities were lower in the AS group than in the A group. This response may be associated with the greater intake of alfalfa hay and NDF in calves fed the control diet, resulting in a larger quantity of undigested substrates entering the hindgut and stimulating microbial fermentation and enzyme production [31]. These findings suggest that starter supplementation altered hindgut fermentation characteristics and nutrient degradation patterns. Nevertheless, dietary starch digestibility and energy metabolism were not directly determined in the present study; therefore, conclusions regarding feed energy utilization efficiency cannot be drawn [32]. Mucosal immune responses are closely associated with gastrointestinal health and microbial colonization during early life. In the present study, starter supplementation increased serum concentrations of IgA, IL-6, TNF-α, M-CSF, and IFN-γ, whereas jejunal TNF-α concentration was lower in the AS group than in the A group. Although IL-6 and TNF-α are generally regarded as pro-inflammatory cytokines, moderate alterations in these cytokines may also reflect immune maturation and host defense development in young ruminants. Previous studies have demonstrated that cytokines such as IL-6 and TNF-α participate in lymphocyte activation, immune regulation, and the establishment of mucosal immunity during early life [33]. Furthermore, interactions between the developing gastrointestinal microbiota and the host immune system are considered essential for the acquisition of immune competence in neonatal ruminants [34]. The concurrent increases in IgA, M-CSF, and IFN-γ observed in the present study suggest enhanced systemic immune competence. In contrast, the reduction in jejunal TNF-α may indicate improved intestinal immune homeostasis and a lower level of local inflammatory stimulation. Collectively, these results suggest that starter supplementation modulated immune function and contributed to the maturation of the gastrointestinal immune system without inducing excessive inflammatory responses.

### 4.3. Intestinal Microbiota

ANOSIM analysis demonstrated that starter supplementation significantly altered the jejunal bacterial community structure, with the most pronounced differences observed at the genus level. In the jejunum, the relative abundances of *Family_XIII_AD3011_group* and *Acetitomaculum* were significantly greater in calves receiving starter feed. Previous studies have suggested that members of *Family_XIII_AD3011_group* may participate in protein and lipid metabolism [35], whereas *Acetitomaculum* can convert carbohydrates into acetate, which may subsequently serve as an energy substrate for the host [36,37]. The enrichment of these genera in the AS group may be associated with the greater crude protein intake and altered nutrient availability resulting from starter supplementation. However, their precise physiological roles in yak calves require further investigation. In contrast, *Olsenella*, *Turicibacter*, *Bacteroides*, *Akkermansia*, and *Colidextribacter* were relatively more abundant in the jejunum of calves from the A group. *Olsenella* has been reported to produce short-chain fatty acids through carbohydrate fermentation [38], suggesting potential differences in substrate utilization between treatments. Previous studies have associated *Turicibacter* and *Colidextribacter* with intestinal inflammatory responses under certain conditions [39], whereas *Bacteroides* and *Akkermansia* are widely recognized as important components of the intestinal microbial ecosystem and have been linked to intestinal barrier integrity and immune regulation [40,41]. Therefore, these microbial alterations should not be interpreted simply as beneficial or detrimental but rather as evidence that starter supplementation modified the ecological composition of the jejunal microbiota. Consistent with these microbial shifts, jejunal TNF-α concentration was lower in the AS group. However, the relationship between microbial community changes and local immune responses remains speculative and warrants further study. In the colon, *Firmicutes* and *Bacteroidota* were the dominant bacterial phyla, consistent with previous observations in ruminants [42]. These phyla play important roles in the degradation and utilization of dietary carbohydrates and proteins [43,44]. The greater relative abundance of *Bacteroidota* and *Bacteroides* in the AS group may be associated with increased crude protein intake following starter supplementation. Members of the genus *Bacteroides* have been reported to possess a broad range of carbohydrate-active enzymes and may contribute to polysaccharide degradation and nutrient turnover within the intestinal ecosystem [45,46,47]. Conversely, *Firmicutes*, *Acetitomaculum*, *Christensenellaceae_R-7_group*, and *Oribacterium* were relatively more abundant in the A group. Because calves in the control group consumed greater amounts of NDF, these differences may reflect variation in the quantity and composition of fibrous substrates entering the hindgut. Similarly, the higher α-amylase and cellulase activities observed in the colon of the A group may be associated with enhanced microbial degradation of undigested dietary components. Previous studies have reported a negative association between *Christensenellaceae* abundance and body weight [48]. In the present study, the lower abundance of *Christensenellaceae_R-7_group* in the AS group coincided with greater final body weight. Nevertheless, these findings represent associations rather than causal relationships, and whether *Christensenellaceae* directly influences growth performance in yak calves remains to be elucidated.

### 4.4. Intestinal Metabolism

Untargeted metabolomic analysis revealed that starter supplementation was associated with substantial alterations in intestinal metabolite profiles. In the jejunum, most of the differential metabolites belonged to organic acids and their derivatives, with several amino acid-related metabolites showing greater abundance in the AS group. KEGG enrichment analysis indicated that pathways related to aminoacyl-tRNA biosynthesis, phenylalanine, tyrosine and tryptophan biosynthesis, alanine, aspartate and glutamate metabolism, and taurine and hypotaurine metabolism were significantly enriched. Among these pathways, L-phenylalanine, L-tyrosine, L-glutamic acid, and L-lysine were enriched in aminoacyl-tRNA biosynthesis. Aminoacyl-tRNA synthesis is a prerequisite for protein translation and cellular metabolic activity. Therefore, the enrichment of amino acid-related metabolites may reflect altered nutrient metabolism in response to starter supplementation. Previous studies have also reported elevated concentrations of L-phenylalanine and L-tyrosine in concentrate-fed yaks compared with forage-fed animals [49], suggesting that these metabolites may be responsive to dietary composition. L-glutamic acid and L-argininosuccinate were enriched in alanine, aspartate, and glutamate metabolism. These metabolites are involved in nitrogen metabolism and energy-related biochemical processes in both host tissues and intestinal microorganisms [50]. In addition, metabolites associated with taurine and hypotaurine metabolism, including L-glutamic acid and cysteine sulfinic acid, were more abundant in the AS group. Because taurine-related pathways have been linked to antioxidant defense mechanisms [51], these changes may indicate alterations in intestinal redox metabolism. Similarly, several glutathione-related metabolites were elevated in the AS group, suggesting a potential association between starter supplementation and antioxidant metabolic processes. However, oxidative stress biomarkers were not directly measured in the present study; therefore, functional conclusions regarding antioxidant capacity cannot be established. In the colon, 5,6-dimethylbenzimidazole and riboflavin were significantly enriched in the AS group. These compounds are involved in vitamin B-related metabolic pathways and may reflect changes in microbial metabolism following dietary intervention [52]. Furthermore, estradiol-related metabolic pathways were enriched in the AS group. Because estrogens have been implicated in immune regulation and intestinal physiology [53], these findings may suggest interactions between nutrient supply, microbial metabolism, and host endocrine responses. Nevertheless, the biological significance of these alterations remains to be clarified. Several metabolites involved in lysine degradation, including 5-aminovaleric acid, N6-acetyl-L-lysine, and L-saccharopine, were also increased in the AS group. These metabolites are intermediates of amino acid catabolism and may indicate enhanced amino acid turnover associated with greater dietary protein intake [54,55]. In addition, alterations in vitamin D metabolites were observed, with cholecalciferol concentrations being higher and calcitriol concentrations being lower in the AS group. Because calcium and phosphorus digestibility were also greater in starter-fed calves, these metabolic differences may be associated with changes in mineral metabolism. However, the regulatory mechanisms involved were not directly investigated and therefore remain speculative. Integrating the microbiome and metabolomic findings, starter supplementation was associated with increased abundances of *Family_XIII_AD3011_group* and *Acetitomaculum* in the jejunum, accompanied by enrichment of several amino acid-related metabolites. These coordinated changes may reflect alterations in nutrient metabolism and microbial ecological function in response to dietary intervention. In the colon, increased abundances of *Bacteroidota* and *Bacteroides* coincided with shifts in metabolites involved in vitamin metabolism and amino acid degradation.

## 5. Conclusions

Our study demonstrates that preweaning starter supplementation reshaped the intestinal microbiota and metabolomic profiles of yak calves and enhanced intestinal immune function. These changes were accompanied by improved nutrient intake and growth performance. Although no significant differences were observed in intestinal morphology, the observed microbial and metabolic alterations suggest that starter supplementation may contribute to gastrointestinal maturation. Therefore, starter supplementation may be considered an effective nutritional strategy for promoting early growth and gastrointestinal health in yak calves.

## Figures and Tables

**Table 1:** Chemical composition of feed ingredients used in the experimental diets (% DM basis).

Items	Milk Replacer (MR)	Alfalfa Hay (AH)
Crude protein	26.60	10.29
Crude fat	19.36	0.80
Neutral detergent fiber	-	58.88
Acid detergent fiber	-	43.92
Calcium	2.50	0.98
Phosphorus	1.40	0.18

**Table 2:** Composition and nutrient levels of starter feed.

Items	Starter Feed
Ingredients (% DM basis)	
Corn, %	46.50
Soybean meal, %	27.00
Wheat bran, %	9.50
Expanded soybean meal, %	8.00
Dried whey, %	5.00
Limestone, %	1.60
Premixture (1), %	1.00
Fat power, %	0.50
CaHPO_4_, %	0.50
NaCl, %	0.40
Nutrient levels	
Crude protein, %	24.10
Ether extract, %	3.74
Neutral detergent fiber, %	10.90
Acid detergent fiber, %	4.10
Ca, %	0.80
P, %	0.45
Ca/P	1.78

Note: (1) The premix provided the following per kg of diets: Fe 22.1 mg, Mn 9.82 mg, Cu 2.25 mg, Zn 27.0 mg, Se 0.19 mg, I 0.54 mg, Co 0.09 mg, VE 4 000 IU, VD 30 000 IU, VA 30 000 IU.

**Table 3:** Feeding schedule and feed allowance of yak calves during the experimental period (g/d per calf, as-fed basis).

Age (d)	A + AS	A	AS	
	MR (g)	AH (g)	AH (g)	SF (g)
45	220	100	50	50
50	240	160	80	80
55	260	200	100	100
60	500	220	110	110
65	560	240	120	120
70	600	280	140	140
75	680	320	160	160
81	680	380	190	190
86	720	420	210	210
91	720	460	230	230
96	720	480	240	240
101	720	480	240	240
106	720	540	270	270
111	720	600	300	300
116	720	700	350	350
121	720	800	400	400
126	680	880	440	440
131	520	920	460	460
137	400	1040	520	520
142	280	1100	550	550

**Table 4:** Effects of starter feeding on growth performance of yak calves.

Items	Group (Mean ± SEM)		*p*-Value
	A	AS	
Dry matter intake of AH (g/d)	639.68 ± 26.38 ^a^	414.05 ± 4.56 ^b^	<0.001
Dry matter intake of SF (g/d)	-	331.35 ± 4.56	-
Dry matter intake (g/d)	1191.08 ± 26.38 ^b^	1296.80 ± 9.13 ^a^	0.001
CP intake (g/d)	224.00 ± 3.67 ^b^	258.03 ± 0.38 ^a^	<0.001
NDF intake (g/d)	371.43 ± 0.06 ^a^	258.30 ± 0.26 ^b^	<0.001
Body weight (kg)	69.30 ± 1.16 ^b^	76.13 ± 1.80 ^a^	0.005
Average daily gain (g)	426.68 ± 8.89	449.23 ± 10.65	0.121
Feed/gain	2.86 ± 0.07	2.96 ± 0.06	0.304
Body height (cm)	78.80 ± 1.00	79.40 ± 1.06	0.685
Body length (cm)	90.70 ± 1.42	88.70 ± 1.11	0.282
Heart girth (cm)	104.70 ± 0.87 ^b^	114.40 ± 0.99 ^a^	<0.001
Cannon circumference (cm)	13.40 ± 0.16 ^b^	14.10 ± 0.18 ^a^	0.010

AH, alfalfa hay; SF, starter feed; CP, crude protein; NDF, neutral detergent fiber; Milk replacer dry matter intake was identical between treatments (551.40 g/d) and is therefore not presented in the table. ^a,b^ within a row with different superscripts means significantly difference. Different letters on the shoulder of the same row of data indicate significant differences (*p* < 0.05).

**Table 5:** Effects of starter feeding on biochemical and immune indexes in plasma of yak calves.

Items	Group (Mean ± SEM)		*p*-Value
	A	AS	
AST (U/L)	54.58 ± 1.21	52.28 ± 3.13	0.513
ALT (U/L)	37.84 ± 2.08	32.34 ± 1.65	0.072
AST/ALT	1.46 ± 0.09	1.62 ± 0.09	0.202
TP (g/L)	65.00 ± 1.05	65.68 ± 0.99	0.651
ALB (g/L)	31.48 ± 0.16 ^a^	30.16 ± 0.40 ^b^	0.016
GLO (g/L)	33.52 ± 1.01	35.52 ± 1.32	0.263
ALB/GLO	0.94 ± 0.03	0.85 ± 0.04	0.112
GLU (mmol/L)	1.35 ± 0.19	1.81 ± 0.21	0.140
CHOL (mmol/L)	2.82 ± 0.19	2.38 ± 0.21	0.154
BUN (mmol/L)	6.20 ± 0.92	6.44 ± 0.46	0.823
CRE (umol/L)	220.40 ± 7.29	216.80 ± 16.12	0.844
TG (mmol/L)	0.38 ± 0.04	0.32 ± 0.03	0.256
LDH (U/L)	654.40 ± 32.66	629.40 ± 23.00	0.549
ALP (U/L)	131.00 ± 10.01	143.40 ± 10.50	0.417
Ca (mmol/L)	2.50 ± 0.05	2.40 ± 0.02	0.122
P (mmol/L)	2.64 ± 0.04	2.63 ± 0.15	0.971
IgA (mg/mL)	2.09 ± 0.09 ^b^	2.60 ± 0.13 ^a^	0.011
IgG (mg/mL)	8.77 ± 0.16	9.16 ± 0.10	0.072
IgM (mg/mL)	1.51 ± 0.06	1.62 ± 0.08	0.329
IL-2 (ng/mL)	1.04 ± 0.06	1.14 ± 0.06	0.262
IL-4 (ng/mL)	0.07 ± 0.00	0.07 ± 0.00	0.071
IL-6 (ng/mL)	0.18 ± 0.01 ^b^	0.22 ± 0.01 ^a^	0.018
IL-10 (ng/mL)	0.07 ± 0.00	0.08 ± 0.00	0.051
TNF-α (ng/mL)	0.15 ± 0.01 ^b^	0.18 ± 0.01 ^a^	0.040
M-CSF (ng/mL)	0.44 ± 0.01 ^b^	0.48 ± 0.01 ^a^	0.030
IFN-α (ng/mL)	0.04 ± 0.00	0.04 ± 0.00	0.479
IFN-β (ng/mL)	0.07 ± 0.00	0.08 ± 0.00	0.082
IFN-γ (ng/mL)	1.45 ± 0.04 ^b^	1.65 ± 0.05 ^a^	0.011

AST, aspartate aminotransferase; ALT, alanine aminotransferase; TP, total protein; ALB, albumin; GLO, globulin; GLU, glucose; CHOL, cholesterol; BUN, blood urea nitrogen; CRE, creatinine; TG, triglyceride; LDH, lactate dehydrogenase; ALP, alkaline phosphatase; Ca, calcium; P, phosphorus; IgA, immunoglobulin A; IgG, immunoglobulin G; IgM, immunoglobulin M; IL-2, interleukin-2; IL-4, interleukin-4; IL-6, interleukin-6; IL-10, interleukin-10; TNF-α, tumor necrosis factor-α; M-CSF, macrophage colony-stimulating factor; IFN-α, interferon-α; IFN-β, interferon-β; IFN-γ, interferon-γ; SEM, standard error of the mean. Values are presented as mean ± SEM. Statistical comparisons between groups were performed using an independent-samples Student’s *t*-test. Means within the same row with different superscript letters (a, b) differ significantly (*p* < 0.05).

**Table 6:** Composition of jejunum and colon bacteria at genus level.

Items (%)	Group (Mean ± SEM)		*p*-Value
	A	AS	
jejunum			
*Romboutsia*	22.46 ± 5.50	14.56 ± 2.65	0.338
*Methanobrevibacter*	7.78 ± 2.42	7.89 ± 2.60	0.975
*Lachnospiraceae_NK3A20_group*	3.99 ± 2.94	7.62 ± 3.88	0.477
*Christensenellaceae_R-7_group*	3.76 ± 0.79	6.77 ± 2.90	0.301
*Family_XIII_AD3011_group*	2.30 ± 0.43 ^b^	5.25 ± 0.30 ^a^	0.004
*Methanosphaera*	2.44 ± 1.26	3.32 ± 1.61	0.681
*Olsenella*	1.38 ± 0.27 ^a^	0.17 ± 0.07 ^b^	0.002
*Acetitomaculum*	0.71 ± 0.17 ^b^	2.10 ± 0.37 ^a^	0.007
*Turicibacter*	0.32 ± 0.07 ^a^	0.07 ± 0.03 ^b^	0.021
*Bacteroides*	0.60 ± 0.10 ^a^	0.22 ± 0.11 ^b^	0.040
*Akkermansia*	0.03 ± 0.01	0.01 ± 0.00	0.070
*Colidextribacter*	0.29 ± 0.04 ^a^	0.10 ± 0.04 ^b^	0.014
colon			
*UCG-005*	14.54 ± 0.52	13.96 ± 1.08	0.644
*Rikenellaceae_RC9_gut_group*	8.43 ± 0.72	10.87 ± 0.77	0.052
*Bacteroides*	6.23 ± 0.96 ^b^	10.17 ± 0.98 ^a^	0.028
*Prevotellaceae_UCG-003*	3.19 ± 0.73	4.01 ± 1.20	0.573
*Alloprevotella*	2.59 ± 0.82	3.71 ± 0.96	0.419
*Christensenellaceae_R-7_group*	2.59 ± 0.35 ^a^	1.72 ± 0.12 ^b^	0.049
*Alistipes*	1.51 ± 0.17	2.26 ± 0.36	0.080
*Romboutsia*	1.81 ± 0.49	1.94 ± 0.33	0.833
*Acetitomaculum*	0.49 ± 0.13 ^a^	0.14 ± 0.03 ^b^	0.042
*Oribacterium*	0.20 ± 0.09 ^a^	0.00 ± 0.00 ^b^	0.044

**Table 7:** KEGG pathway enrichment analysis of differential metabolites in the jejunum.

Items	Differential Metabolites
Aminoacyl-tRNA biosynthesis	L-Phenylalanine ↑, L-Tyrosine ↑, L-Glutamic acid ↑, L-Lysine ↑
Phenylalanine, tyrosine and tryptophan biosynthesis	L-Phenylalanine ↑, L-Tyrosine ↑ D-Erythrose 4-phosphate ↑
Alanine, aspartate and glutamate metabolism	L-Glutamic acid ↑, L-Argininosuccinate ↑
Taurine and hypotaurine metabolism	L-Glutamic acid ↑, L-Cysteinesulfinic acid ↑
Glutathione metabolism	L-Pyroglutamic acid ↑, L-Glutamic acid ↑, Spermine ↑
alpha-Linolenic acid metabolism	13 (S)-HOTrE ↓, Jasmonic acid ↓

Note: ↑: the arrow represents the metabolite up-regulated in the AS group; ↓: the arrow represents the metabolite down-regulated in the AS group.

**Table 8:** KEGG pathway enrichment analysis of differential metabolites in the colon.

Items	Differential Metabolites
Riboflavin metabolism	5,6-Dimethylbenzimidazole ↑, Riboflavin ↑
Endocrine resistance	Estradiol ↑, Androstenedione ↓
Lysine degradation	5-Aminovalericacid ↑, 6-N6-Acetyl-L-lysine ↑, L-Saccharopine ↑
Steroid biosynthesis	Calcitriol ↓
Endocrine and other factor-regulated calcium reabsorption	Calcitriol ↓
Tuberculosis	Calcitriol ↓

Note: ↑ indicates metabolites up-regulated in the AS group; ↓ indicates metabolites down-regulated in the AS group.

## Data Availability

The data supporting the findings of this study are available from the corresponding author upon reasonable request.

## Abbreviations

The following abbreviations are used in this manuscript:

A	control group
ALB	albumin
ALP	alkaline phosphatase
ALT	glutamic-pyruvic transaminase
AS	experimental group
AST	glutamic-oxaloacetic transaminase
BUN	blood urea nitrogen
Ca	calcium
CHOL	cholesterol
CRE	creatinine
GLO	globulin
GLU	glucose
IFN-γ	interferon γ
IgA	immunoglobulin A
IL-6	interleukin 6
LDH	lactic dehydrogenase
M-CSF	macrophage colony stimulating factor
P	phosphorus
TG	triglyceride
TNF-α	tumor necrosis factor α
TP	total protein
